# The Outcomes of Colorectal Endoscopic Submucosal Dissection in Patients with Chronic Kidney Disease: A Honam Association for the Study of Intestinal Disease (HASID) Multicenter Study

**DOI:** 10.3390/diagnostics14131459

**Published:** 2024-07-08

**Authors:** Byung Chul Jin, Dong Hyun Kim, Geom-Seog Seo, Sang-Wook Kim, Hyung-Hoon Oh, Hyo-Yeop Song, Seong-Jung Kim, Young-Eun Joo, Jun Lee, Hyun-Soo Kim

**Affiliations:** 1 Department of Internal Medicine, Research Institute of Clinical Medicine, Biomedical Research Institute, Jeonbuk National University Medical School, Jeonbuk National University, Jeonju 54907, Republic of Korea; 23743@jbuh.co.kr; 2 Department of Internal Medicine, Medical School, Chonnam National University, Gwangju 61469, Republic of Korea; bono343@cnuh.com (D.H.K.); hyung1125@naver.com (H.-H.O.); yejoo@chonnam.ac.kr (Y.-E.J.); dshskim@jnu.ac.kr (H.-S.K.); 3 Department of Internal Medicine, Digestive Diseases Research Institute, School of Medicine, Wonkwang University, Iksan 54538, Republic of Korea; bfsongc@hanmail.net; 4 Department of Internal Medicine, College of Medicine, Chosun University, Gwangju 61452, Republic of Korea; ygegh@chosun.ac.kr (S.-J.K.); leejun@med.chosun.ac.kr (J.L.)

**Keywords:** chronic kidney failure, colon, colorectal neoplasm, endoscopic submucosal dissection, endoscopy

## Abstract

Colorectal neoplasms are prevalent in patients with chronic kidney disease (CKD); however, the safety and efficacy of colorectal endoscopic submucosal dissection (ESD) are not well understood. This retrospective analysis included ESD procedures performed in 1266 patients with CKD across five tertiary medical institutions from January 2015 to December 2020. Patients were categorized based on their estimated glomerular filtration rate (eGFR), which ranged from CKD1 to CKD5 (including those on dialysis). We found that en bloc resection rates remained high across all CKD stages, affirming the procedural efficacy of ESD. Notably, the prevalence of cardiovascular comorbidities, such as ischemic heart disease and diabetes mellitus, significantly increased with an advancing CKD stage, with a corresponding increase in the Charlson Comorbidity Index, highlighting the complexity of managing these patients. Despite these challenges, the complete resection rate was lower in the CKD5 group (50%) than in the CKD1 group (83.4%); however, procedural complications, such as perforation and bleeding, did not significantly differ among the groups. The predictive models for complete resection and major complications showed no significant changes with a decreasing eGFR. These findings underscore that ESD is a feasible and safe treatment for colorectal neoplasms in patients with CKD, successfully balancing the inherent procedural risks with clinical benefits.

## 1. Introduction

Colonoscopy remains the gold standard for diagnosing colon cancer, significantly enhancing the early detection of this leading cause of cancer-related mortality worldwide [1]. The increased prevalence of colonoscopy has been instrumental in increasing the detection rate of this malignancy, thereby playing a crucial role in early intervention and improving patient outcomes. Notably, the increasing adoption of advanced endoscopic techniques, such as endoscopic mucosal resection and endoscopic submucosal dissection (ESD), for treating premalignant and early-stage colonic lesions has exemplified the significant advancements in medical technology [2]. These procedures aim to balance therapeutic efficacy with minimal invasiveness and offer significant benefits over traditional surgical methods [3].

Endoscopic procedures are gaining popularity, particularly among the elderly and patients with multiple comorbidities, such as chronic kidney disease (CKD) [4]. With over 10% of the global population, approximately 800 million individuals, being affected by CKD, the demand for less invasive yet effective treatment modalities is increasing globally. This shift is driven by the prevalence of CKD and the necessity for patient-centered care in CKD management, which seeks to reduce invasiveness while ensuring treatment efficacy [5]. The interplay between CKD and cancer risk is significant; patients with CKD have a heightened risk of developing colon cancer owing to shared risk factors and the exacerbating effects of renal dysfunction on cancer progression. This underscores the critical need for vigilant oncological screening and effective interventions for this demographic [6,7,8,9].

Moreover, patients with CKD, particularly those undergoing dialysis, experience a significantly higher rate of complications from endoscopic procedures, such as bleeding. These patients face a 1.5 times greater risk of such complications, which are amplified by the platelet dysfunction associated with dialysis [10,11]. The high mortality and complication rates associated with surgical procedures in patients with CKD underscore the need for safer and less invasive alternatives. Although ESD is less invasive than traditional surgical resection, it carries its own set of risks, notably post-procedural complications. The current literature lacks sufficient data on the outcomes following ESD, especially concerning the risk of bleeding in patients with CKD [12,13]. This gap in the literature indicates a critical need for comprehensive studies that can offer insights into the procedural risks and benefits that are specific to patients with CKD. This study aims to fill this gap. 

## 2. Materials and Methods

### 2.1. Study Design and Patients

This multicenter retrospective study included data collected between January 2015 and December 2020. Data were obtained from five tertiary medical centers in Honam Province, South Korea (Chonnam National University Hospital, Chonnam National University Hwasun Hospital, Jeonbuk National University Hospital, Chosun University Hospital, and Wonkwang University Hospital). The study protocol was approved by the Institutional Review Boards of Chonnam National University Hospital (CNUH-2022-060), Chonnam National University Hwasun Hospital (CNUHH-2022-208), Chosun University Hospital (CHOSUN 2022-03-006-001), Jeonbuk National University Hospital (CNU 2022-12-057), and Wonkwang University Hospital (WKUH 2022-10-007). The requirement for informed consent was waived. 

### 2.2. Inclusion and Exclusion Criteria

#### 2.2.1. Inclusion Criteria

The inclusion criteria involved patients who were diagnosed with CKD stages 1–5, patients who underwent colorectal ESD between January 2015 and December 2020, patients with an eGFR of <90 mL/min/1.73 m^2^, patients aged 18 and above, and patients who were included irrespective of the number of previous ESD procedures. 

#### 2.2.2. Exclusion Criteria

The exclusion criteria included patients with incomplete medical records, patients who underwent ESD for reasons other than colorectal neoplasms, patients with diagnoses of other solid tumors, patients who had undergone prior colorectal surgeries, and patients who had previously received chemotherapy.

#### 2.2.3. ESD Indication

ESD was primarily performed for colorectal neoplasms, including adenomas, intramucosal cancer, and early-stage invasive cancer.

#### 2.2.4. Medication and Chemotherapy

This study also considered patients’ medication histories, including the use of anticoagulants and antiplatelets. Patients were required to discontinue anticoagulant and antiplatelet therapies based on endoscopic resection guidelines. Patients who had previously undergone chemotherapy were excluded from the analysis.

#### 2.2.5. Primary Colorectal Cancers

Only primary colorectal cancers were included in this analysis. 

### 2.3. Data Collection and Outcomes

Demographic and procedural data, including variables such as age, sex, lesion location, gross morphology, lesion size, histopathological findings, adverse events, and rate of complete resection, were collected from electronic medical records. Lesions were classified into the following categories according to the Paris classification: protruding, superficial and elevated, flat, and flat and depressed [14]. The lesion locations were divided into three regions: the right colon (including the cecum, ascending colon, and transverse colon), left colon (including the descending colon and sigmoid colon), and rectum. En bloc resection entailing a single-piece excision of the complete lesion was performed meticulously. Pathological analyses were conducted to determine the presence of margins and establish the final diagnosis, and lesion sizes were assessed endoscopically.

Anticoagulant and antiplatelet therapies were discontinued in accordance with the endoscopic resection guidelines, taking into account any patient-specific thromboembolic risk factors and bleeding potential [15]. Sedation was achieved using midazolam and/or propofol with pethidine added as necessary while maintaining a constant oxygen supply of 2 L/min through the nasal cavity. Sedation levels were adjusted based on the continuous monitoring of vital signs, including blood pressure, heart rate, and oxygen saturation. Hypotension and oxygen desaturation were defined as a systolic blood pressure of less than 90 mmHg and arterial oxygen saturation <90% for at least 10 s, respectively.

Procedure-related complications, such as bleeding requiring transfusions or emergency endoscopy; a drop in hemoglobin levels of >2 g/dL; and perforation, identified by an endoscopic visualization of peritoneal fat or intra-abdominal organs or by imaging, were defined [16]. Post-ESD coagulation syndrome (PECS) is characterized by signs of post-procedural inflammation, including abdominal tenderness, fever, leukocytosis, elevated C-reactive protein levels, and the absence of evidence of perforation. The major complications included procedure-related bleeding, perforation, and PECS [17].

### 2.4. Statistical Analysis

Continuous variables were presented as either the mean ± standard deviation or median with an interquartile range, while categorical variables were presented as frequency counts and percentages. A comparative analysis of the four study cohorts was conducted. For continuous data, the analysis employed Student’s *t*-test or the Mann–Whitney *U* test, based on data distribution appropriateness. Categorical variables were evaluated using the chi-square or Fisher’s exact tests, as indicated by the data characteristics. Special emphasis was placed on contrasting the middle-aged cohort data with those of the other groups.

To evaluate the relationship between eGFR and the procedural outcomes, we employed restricted cubic splines with three knots to model the likelihood of complete resection and major complications and enhance the model’s flexibility. This approach allowed for a flexible and nonlinear examination of the effects of declining renal function on the procedural outcomes. Graphical representations of these models were generated to illustrate the predicted probabilities and 95% confidence intervals (CIs) for complete resection and major complications at varying eGFR levels. These predictive models were developed to identify significant predictors of complete resection and major complications. These models incorporated variables such as age, lesion size, lesion location, histopathological findings, and CKD stage. The models were evaluated using goodness-of-fit measures. 

All statistical analyses were performed using SPSS version 25 (IBM Corp., Armonk, NY, USA) and Stata/SE 16.1 (StataCorp, College Station, TX, USA). Statistical significance was set at *p* < 0.05 for all analyses. This study adhered to rigorous statistical standards to ensure the reliability and validity of the findings.

## 3. Results

### 3.1. Baseline Characteristics of Patients with CKD 

In this retrospective cohort study, we assessed the baseline characteristics of 1266 patients with CKD undergoing ESD for colorectal neoplasms. The age distribution revealed a progressive increase in the mean age from 62.4 ± 11.2 years in early-stage CKD1 to 65.7 ± 6.6 years in advanced CKD5. This trend towards older age was correlated with advancing stages of kidney disease. Although the size of the colorectal neoplasm specimens showed a slight increase from 29.3 ± 12.3 mm in the CKD1 group to 35.3 ± 21.6 mm in the CKD5 group, these variations were not statistically significant across the CKD stages, indicating consistent lesion sizes regardless of renal function impairment.

**Figure 1:**
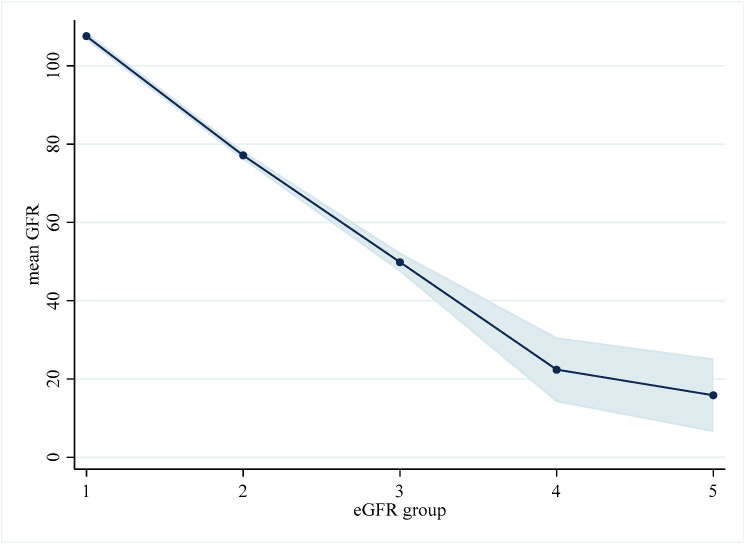
Decline of mean estimated glomerular filtration rate (eGFR) across chronic kidney disease (CKD) stages. The shaded area represents the 95% confidence intervals (CIs) for the estimated glomerular filtration rates. eGFR, estimated glomerular filtration rate.

The delineation of the CKD stages showed a stark contrast in the mean eGFRs. The mean eGFR commenced at a high value of 107.60 in the CKD1 group and descended steeply to a markedly diminished value of 15.85 in the CKD5 group. The intermediate stages displayed mean eGFRs of 77.15 (95% CI: 76.08–78.22) in the CKD2 group, 49.86 (95% CI: 47.42–52.31) in the CKD3 group, and 22.39 (95% CI: 14.15–30.63) in the CKD4 group, with each successive stage demonstrating an accelerating decline. The widening of the 95% CIs at the advanced stages indicates an increasing variability in eGFR measurements. Figure 1 illustrates the progressive deterioration of eGFR associated with CKD and the variability in measurements (Figure 1). 

We observed a significant increase in the prevalence of major comorbidities, such as ischemic heart disease and diabetes mellitus, with advancing CKD stages. Diabetes with end-organ damage showed a tenfold increase from 1.0% in the CKD1 group to 14.3% in the CKD5 group. Cerebrovascular accidents also increased sharply from 0.3% to 14.3% from the earliest to the latest stages of CKD. Aspirin and clopidogrel intake were inversely related to CKD stage, while warfarin usage increased substantially with increasing CKD stages. The Charlson Comorbidity Index paralleled the CKD progression, being amplified from a value of 0.8 ± 1.1 in the CKD1 group to 6.0 ± 2.2 in the CKD5 group.

The distribution of tumor locations exhibited a symmetrical pattern between the right and left sides of the colon across the CKD stages, with no significant differences in the prevalence of rectal tumors as CKD progressed. Morphologically, the majority of neoplasms were of the protruding type, with flat-type neoplasms being the least prevalent in the CKD5 group. Histologically, adenomas were the predominant finding across all CKD stages; however, their prevalence decreased as renal impairment progressed, resulting in a higher incidence of invasive cancers at the more advanced stages. The incidence of invasive cancers rose with the progression of CKD (Table 1).

### 3.2. Treatment Outcomes

When assessing the outcomes of ESD in patients with CKD, the en bloc resection rates were high across all stages, with a notable achievement rate of 100% in the CKD5 cohort. The consistently high rate of complete resection peaked at 88.2% in the CKD3 group. The complication rates remained low, with infrequent occurrences of perforations (0.7%), which were most commonly observed in the CKD1 group. However, bleeding complications significantly increased in the CKD5 group (14.3%, *p* < 0.05).

The occurrence of post-procedure preventive measures, such as PECS, was uniformly low. Sedation practices differed based on CKD severity, with an increased use of midazolam and propofol observed in the intermediate stages (*p* < 0.01). Histologically, adenomas were the most common finding, particularly in the CKD1 group (54.6%), with no significant variation in their distribution in later stages (Table 2).

### 3.3. Predictive Models for the Rate of Complete Resection and Complications

In the predictive model analysis, we examined the outcomes of ESD in relation to renal function, indicated by eGFR metrics. The analysis of our dataset revealed a consistent rate of complete resection across varying eGFR levels. This trend is noteworthy, as it suggests that the efficacy of ESD procedures is maintained even as the renal function declines, which is a common complication associated with progressive CKD. These results underscore the reliability of ESD as a treatment for colorectal neoplasms, regardless of the CKD stage.

**Figure 2:**
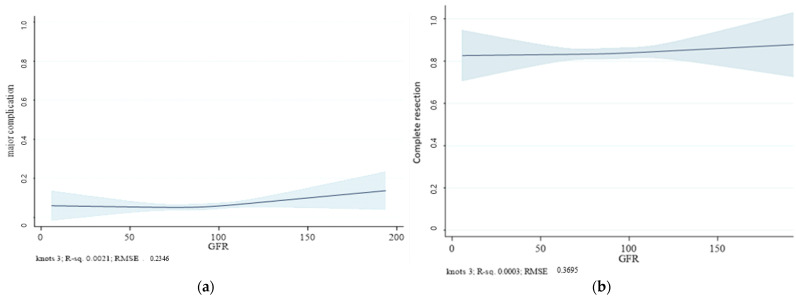
Predictive margins for endoscopic submucosal dissection outcomes relative to eGFR. (**a**) Predictive margin for the rate of complete resection against eGFR, indicating a stable likelihood of complete resection across a range of renal functions. (**b**) Predictive margin for major complications against eGFR, demonstrating that major complications do not significantly increase as renal function declines. The shaded area represents the 95% confidence intervals (CIs) for the estimated major complication rates. GFR, glomerular filtration rate; RMSE, root mean square error.

The rates of major complications remained consistently low across the mild-to-moderate stages of CKD. Although there was a slight increase in major complications among patients with advanced renal failure, this increase was not statistically significant. This indicated that the overall incidence of major complications remained low, even as the severity progressed. This bidirectional analysis confirmed the potential of ESD as an effective and safe intervention for colorectal neoplasm treatment in patients with CKD, providing support for its application in this high-risk patient population (Figure 2).

## 4. Discussion

The safety of colorectal ESD in patients with CKD is a critical concern given the increased vulnerability of this patient population [18]. Our analysis focused on evaluating the safety profile across different stages of renal impairment to provide a nuanced understanding of the impact of the procedure. Traditional surgical interventions in patients with CKD can be particularly challenging because of their underlying renal conditions, which are associated with higher mortality rates and prolonged hospital stays compared to patients without CKD [19,20,21]. In contrast, less invasive endoscopic treatments, such as ESD, offer a viable alternative [22]. By minimizing physical trauma and the potential for adverse effects that is often associated with general surgery, ESD enables the effective management of early-stage colorectal cancer, while potentially reducing postoperative complications and hospitalization duration.

Our large-scale study revealed consistently high en bloc resection rates across all CKD stages with a remarkable achievement rate of 100% in the CKD5 cohort. This suggests that ESD maintains procedural efficacy, even in patients with severe renal dysfunction, highlighting its reliability as a treatment option [23]. The high success rate of en bloc resection in patients with CKD, especially in those with advanced-stage renal impairment, underscores the potential of ESD as a reliable treatment modality. Despite the challenges posed by their health status and associated comorbidities, complete resection rates remained substantial, particularly in the CKD3 group, illustrating the efficacy of the procedure in achieving oncological completeness without compromising safety. These findings indicate that ESD is a valuable and effective treatment strategy that can achieve successful resection in patients with varying degrees of renal impairment.

Furthermore, while our study observed low overall rates of procedural complications, such as perforation and bleeding, the nuanced manifestations of risk among patients with CKD warrant special attention [24,25]. Despite the relatively low incidence of major bleeding events across the CKD stages, patients undergoing dialysis exhibited a higher tendency towards bleeding due to platelet dysfunction and other coagulation abnormalities that are inherent to end-stage renal disease [11]. This is consistent with findings from the existing literature that highlight the elevated risk of bleeding in patients with advanced CKD [26].

Although bleeding complications remain rare overall, their increased frequency in the CKD5 group necessitated heightened clinical vigilance and proactive management to preemptively address this elevated risk. The observed increase in bleeding risk underscores the importance of meticulous patient monitoring and tailored procedural planning [27]. Special precautions must be taken to mitigate these risks, including a preprocedural assessment of the coagulation status and careful management of anticoagulant medications [28]. In addition to bleeding, the association between CKD and the occurrence of perforation or PECS has not been well studied. In this large study, we found that the incidences of PECS and perforation were not particularly high in the high-eGFR group. This was reflected in the prediction model, which showed that as the eGFR decreased, the occurrence of major complications did not increase significantly. Few sedation-related complications were reported, indicating that the use of sedative medications in patients with advanced CKD is not a significant barrier to performing colorectal ESD.

Given the elevated procedural risks, establishing a safe environment for ESD in patients is imperative [20]. This includes not only technical preparation and skill but also a comprehensive preoperative evaluation to identify any potential risks that could compromise procedure safety. By implementing such targeted strategies, healthcare providers can ensure that ESD remains a viable and effective treatment option for colorectal neoplasms in patients with CKD, despite their increased vulnerability to complications [29]. 

In addition to our analyses, we developed a predictive model using restricted cubic splines to explore the relationship between the CKD stage and colorectal ESD outcomes. This modeling technique enabled a nuanced examination of the nonlinear relationships between renal function deterioration, as indicated by a declining eGFR, and key procedural outcomes, such as complete resection rates and major complication incidences [23]. Employing this model, we observed that the rates of complete resection remained stable across varying degrees of renal impairment until a marked decline in patients with eGFR below 30 mL/min/1.73 m^2^ was observed, mirroring trends seen in other studies, where the procedural efficacy decreases as the patient’s condition deteriorates. The rates of major complications remained consistently low across the mild-to-moderate stages of CKD [25]. Although there was a slight increase in major complications among patients with advanced renal failure, this increase was not statistically significant or substantial enough to be clinically alarming. This indicates that despite the increased complexity of managing patients with severe CKD, ESD can still be performed safely with careful patient monitoring and selection [30]. These findings support the notion that ESD remains a safe option for patients at various stages of CKD, balancing procedural risks with clinical benefits.

Although this study provided valuable insights into the efficacy and safety of colorectal ESD in patients with CKD, it had several limitations. First, as a retrospective analysis, it lacked the level of data quality and control over variables offered by prospective studies. This limitation could have affected the reliability and depth of the findings compared with those obtained from a prospectively designed study with tighter parameter controls. Second, the findings of the study may have limited applicability beyond the East Asian population, primarily South Korea, potentially affecting its generalizability to other ethnic and regional groups. The focus on a single ethnic group and regional healthcare setting might limit the applicability of the findings to populations of different ethnic backgrounds or healthcare systems. Third, variations in the expertise and techniques of operating physicians across multiple institutions could have introduced additional bias, impacting the study outcomes. Fourth, the relatively small number of patients in the CKD5 group (only 7 of 1266) limited the robustness of the analysis for this subgroup. This small sample size reduced the statistical power and may have affected the generalizability of the results to patients with severe CKD, thereby necessitating further studies with larger CKD5 populations to validate these findings. Lastly, the study did not record whether it was the first ESD for all patients. This specific detail was not included, and its absence could have implications for the interpretation of the procedural outcomes and risks. Future studies should consider including this factor to enhance the comprehensiveness of the analysis.

Despite these limitations, the multicenter design of this study ensured a robust analysis by including a large and diverse patient cohort, thus enhancing the statistical power and applicability of the findings across various CKD stages. The results of this study contribute to a better understanding of how CKD stages influence procedural safety and effectiveness, underscoring the need for targeted procedural strategies to manage the increased risk in this vulnerable patient group.

## 5. Conclusions

We found that colorectal ESD is a feasible and safe procedure for patients with CKD across all stages. Despite the increased complexity and comorbidity burden in advanced CKD stages, ESD maintained high en bloc and complete resection rates with low complication rates, demonstrating its reliability and effectiveness. These findings underscore the potential of ESD as a valuable treatment modality for colorectal neoplasms in patients with CKD, providing a less invasive alternative to traditional surgical methods with significant clinical benefits.

## Figures and Tables

**Table 1:** Baseline characteristics across different stages of CKD.

	Total (*n* = 1266)	CKD1 (*n* = 610)	CKD2 (*n* = 538)	CKD3 (*n* = 102)	CDK4 (*n* = 9)	CKD5 (*n* = 7)
Age, years, mean ± SD	65.4 ± 11.2	62.4 ± 11.5	67.1 ± 10.1	74.0 ± 8.0	70.4 ± 11.6	65.6 ± 7.6
Female sex, *n* (%)	510 (40.3)	276 (45.2)	192 (35.8)	35 (34.3) *	4 (44.5)	3 (42.9)
Specimen size, long axis (mm), mean ± SD	29.9 ± 12.4	29.3 ± 12.3	30.1 ± 11.5	32.5 ± 15.6	29.4 ± 14.0	35.3 ± 21.6
History, *n* (%)						
Ischemic heart disease	51 (4.0)	19 (3.1)	22 (4.1)	7 (6.9)	0	3 (42.9) **
Chronic heart failure	17 (1.3)	2 (0.3)	6 (1.1)	7 (6.9) *	1 (11.1) **	1 (14.3) **
Diabetes mellitus	252 (19.9)	97 (15.9)	110 (20.4) *	38 (37.3) **	4 (44.4) *	3 (42.9) *
Diabetes mellitus with end-organ disease	16 (1.5)	1 (0.2)	3 (0.6)	3 (2.9) **	4 (44.4) **	5 (71.4) **
Cerebrovascular accident	38 (3.0)	11 (1.8)	18 (3.3)	6 (5.9) **	3 (33.3) **	0
Medication, *n* (%)						
Aspirin	106 (8.4)	32 (5.2)	54 (10.0) **	13 (12.7) **	3 (33.3) **	4 (57.1) **
Clopidogrel	41 (3.2)	11 (1.8)	20 (3.7) *	8 (7.8) **	0	2 (28.6) **
Warfarin	5 (0.4)	0	4 (0.7) *	1 (1.0) **	0	0
Charlson Comorbidity Index, mean ± SD	1.0 ± 1.3	0.8 ± 1.1	1.0 ± 1.2 **	1.8 ± 1.6 **	3.7 ± 2.3 **	6.0 ± 2.2 **
Tumor location, *n* (%)						
Right side	649 (51.3)	316 (51.8)	271 (50.4)	55 (53.9)	3 (33.3)	4 (57.1)
Left side	302 (23.9)	151 (24.8)	127 (23.6)	21 (20.6)	2 (22.2)	1 (14.3)
Rectum	315 (24.9)	143 (23.4)	140 (26.0)	26 (25.5)	4 (44.4)	2 (28.6)
Morphology, *n* (%)						
Protruding	256 (20.2)	106 (17.4)	114 (21.2)	28 (27.5)	3 (33.3)	5 (71.4) **
Superficial, elevated	987 (78.0)	493 (80.8)	414 (77.0)	72 (70.6)	6 (66.7)	2 (28.6) **
Flat	13 (1.0)	7 (1.1)	4 (0.7)	2 (2.0)	0	0
Flat, depressed	10 (0.8)	4 (0.7)	6 (1.1)	0	0	0
Method of sedation, *n* (%)						
Midazolam	816 (64.5)	403 (66.1)	328 (61.0)	72 (70.6) **	7 (77.8)	6 (85.1)
Propofol	5 (0.4)	3 (0.5)	2 (0.4)	0	0	0
Midazolam and propofol	65 (5.1)	31 (5.1)	26 (4.8)	7 (6.9) **	1 (11.1)	0
Histologic findings, *n* (%)						
Adenoma	665 (52.5)	333 (54.6)	279 (51.9)	50 (49.0)	3 (33.3)	0
Intramucosal cancer	263 (20.8)	115 (18.9)	120 (22.3)	22 (21.6)	2 (22.2)	4 (57.1) **
Invasive cancer	338 (26.7)	162 (26.6)	139 (25.8)	30 (29.4)	4 (44.4)	3 (42.9) **
Hospital stay (day), mean ± SD	4.4 ± 2.3	4.5 ± 2.4	4.3 ± 1.7	4.4 ± 3.6	4.3 ± 1.6	6.1 ± 4.5

CKD groups are categorized into five groups based on eGFR. CKD, chronic kidney disease; SD, standard deviation. * *p* < 0.05 compared with the CKD 1 group; ** *p* < 0.01 compared with the CKD 1 group.

**Table 2:** Treatment outcomes across different stages of CKD.

	Total (*n* = 1266)	CKD1 (*n* = 610)	CKD2 (*n* = 538)	CKD3 (*n* = 102)	CDK4 (*n* = 9)	CKD5 (*n* = 7)
En bloc resection, *n* (%)	1131 (89.3)	539 (88.4)	486 (90.3)	91 (89.2)	8 (88.9)	7 (100.0)
Complete resection, *n* (%)	1060 (83.7)	510 (83.6)	449 (83.5)	90 (88.2)	6 (66.7)	5 (71.4)
Perforation, *n* (%)	9 (0.7)	6 (1.0)	3 (0.6)	0	0	0
Bleeding, *n* (%)	30 (2.4)	16 (2.6)	9 (1.7)	4 (3.9)	0	1 (14.3)
PECS, *n* (%)	37 (2.9)	24 (3.9)	12 (2.2)	1 (1.0)	0	0
Sedation-related complication, *n* (%)						
Hypoxemia	0	0	0	0	0	0
Hypotension	1 (0.1)	1 (0.2)	0	0	0	0

CKD groups are categorized into five groups based on eGFR. CKD, chronic kidney disease; PECS, post-endoscopic submucosal dissection coagulation syndrome.

## Data Availability

The data are not publicly available due to privacy and ethical restrictions. Data presented in this study are available upon request from the corresponding author.
